# Happy Being Me: outcomes of a peer-based body dissatisfaction prevention intervention in young adolescent girls

**DOI:** 10.1186/2050-2974-1-S1-O33

**Published:** 2013-11-14

**Authors:** Sian McLean, Susan Paxton, Eleanor Wertheim

**Affiliations:** 1 School of Psychological Science, La Trobe University, Australia

## 

This study aimed to examine body dissatisfaction and risk factor outcomes following participation in Happy Being Me, a 6-lesson peer-based prevention intervention for young adolescent girls. Participants were 491 female year 7 students randomly allocated to the Happy Being Me intervention condition (N=295) or control condition (N=196). Self-report questionnaire data was collected at baseline, post-program, and 6- and 12-month follow-up. Preliminary data analyses for the incomplete sample indicate baseline to 6-month follow-up improvements for internalisation of the thin ideal (F = 5.38 (1, 332), p = .021), appearance comparisons (F = 10.21 (1, 312), p = .002), and media literacy (F = 15.98 (1, 327), p < .001) in the Happy Being Me intervention condition relative to the control condition. Reductions in the intervention condition were not significantly different from the control condition for weight and shape concern (F = 0.28 (1, 313), p = .599) or body dissatisfaction (F = 2.48 (1, 313), p = .116). Twelve-month follow-up data will be presented for the complete sample. The results from this study provide preliminary evidence for positive outcomes following participation in a classroom delivered multi-component peer-based body dissatisfaction prevention intervention.

This abstract was presented in the **Prevention** stream of the 2013 ANZAED Conference.

